# Supplementary material for: FLI1 promotes IFN-γ-induced kynurenine production to impair anti-tumor immunity
Source: Nat Commun. 2024 May 30;15:4590. doi: 10.1038/s41467-024-48397-9 (PMC11139667; doi:10.1038/s41467-024-48397-9)
Supplement: Supplementary file 1 — Supplementary Information [file 41467_2024_48397_MOESM1_ESM.pdf]

Supplementary Information

FLI1 promotes IFN-γ-induced kynurenine production to impair anti-tumor immunity

Chen et al.

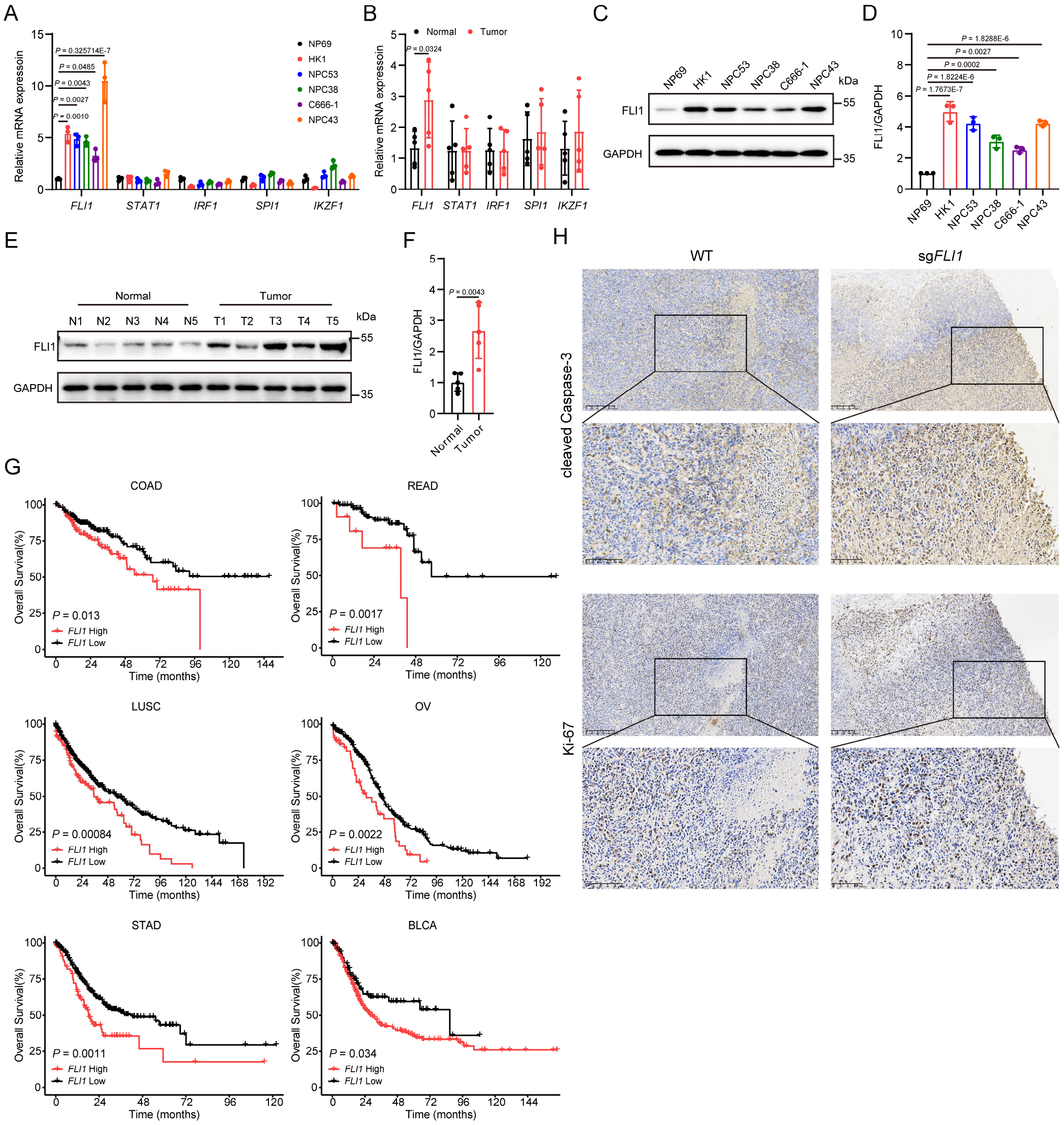

Supplementary Figure 1. (Extended data related to Figure 1)

(A) *FLI1*, *STAT1*, *IRF1*, *SPI1* and *IKZF1* mRNA levels in normal nasopharyngeal epithelial and NPC cell lines. **(B)** *FLI1*, *STAT1*, *IRF1*, *SPI1* and *IKZF1* mRNA level in normal nasopharynx tissues and NPC tissues. **(C and D)** FLI1 protein level in normal nasopharyngeal epithelial and NPC cell lines. **(E and F)** FLI1 protein level in normal nasopharynx tissues and NPC tissues. The data are presented as the mean ± SD **(A, B, D and F)**. The results are representative of three independent experiments **(A and D)**. Statistical analysis was performed by one-way ANOVA with Tukey multiple comparisons test **(A and D)** and two-tailed unpaired *t* test **(B and F)**. **(G)** Kaplan-Meier survival analysis of the association between overall survival and the expression levels of FLI1 across patient cohorts with COAD, READ, LUSC, ovarian cancer (OV), stomach cancer (STAD) and

bladder urothelial carcinoma (BLCA) as sourced from the TCGA database. Statistical analysis was performed by log-rank test. **(H)** Representative images of cleaved caspase3- and Ki67- positive cells as analyzed by IHC staining. *n* = 6 for each group. Scale bars, 200 μm (upper panel), 100 μm (lower panel). Source data are provided as a Source Data file.

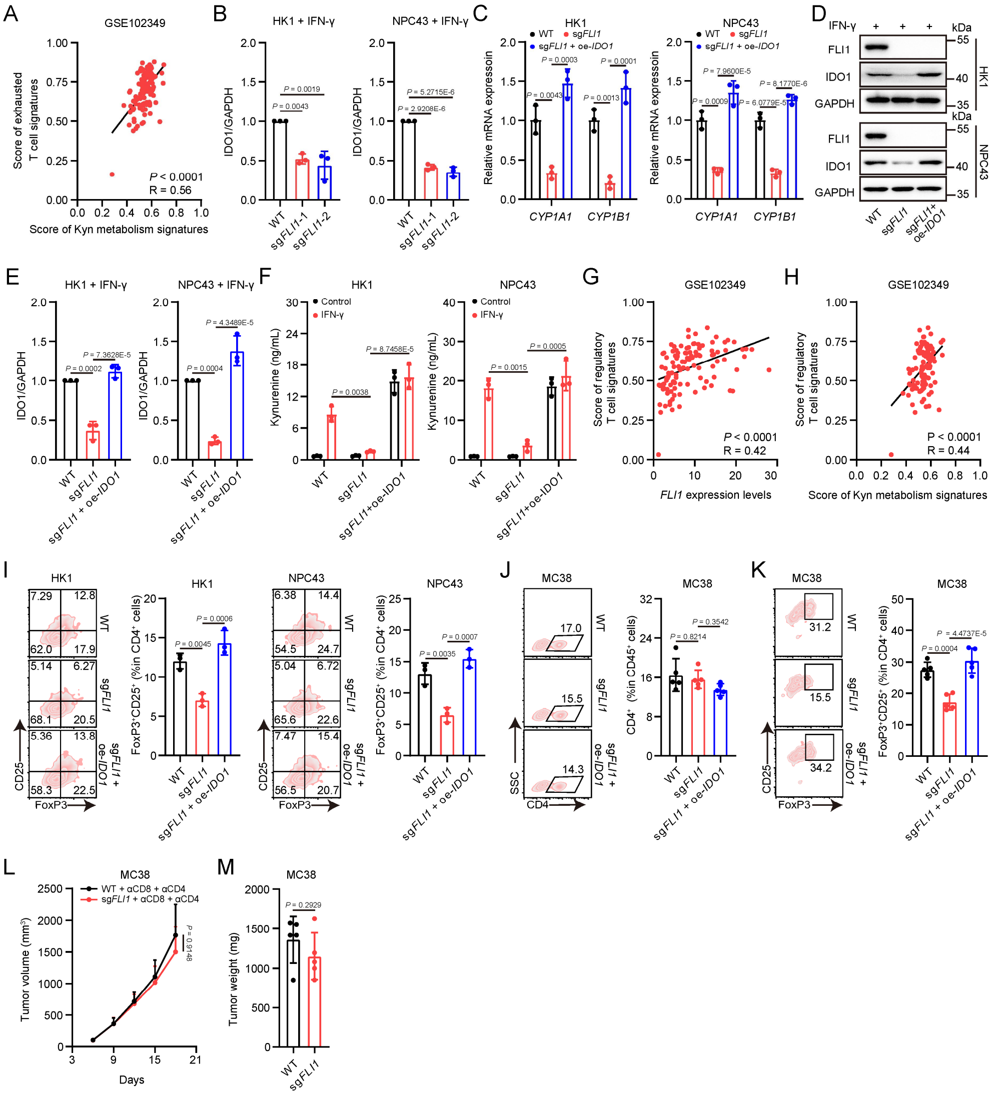

Supplementary Figure 2. (Extended data related to Figure 2)

(A) Analysis of the correlation between the Kyn metabolism signatures and the exhausted T cell signatures in NPC. **(B)** Effect of *FLI1* KO on IDO1 protein expression under IFN-γ treatment in HK1 and NPC43 cells. **(C)** The indicated NPC cells were cocultured with activated human T cells for 48 hours. *CYP1A1* and *CYP1B1* mRNA levels in T cells were detected. **(D and E)** IDO1 protein level in the indicated NPC cells was detected. Cells were treated with IFN-γ for 24 hours. **(F)** Kyn production in the culture supernatants of the indicated NPC cells was determined. **(G)** Analysis of the correlation between the *FLI1* expression level and the regulatory T cell signatures. **(H)** Analysis of the correlation between the Kyn metabolism signatures and the regulatory T cell signatures. **(I)** The indicated NPC cells were cocultured with activated human $CD4^+$ T cells for 48h. The proportion of $CD25^+$ $FoxP3^+$ cells among $CD4^+$ T cells was determined by flow cytometry. The results are

representative of three independent experiments **(B-F and I)**. The data are presented as the mean ± SD **(B, C, E, F and I)**. Statistical analysis was performed by one-way ANOVA with Tukey multiple comparisons test **(B, C, E, F and I)** and two-sided χ^2 test **(A, G and H)**. **(J and K)** WT, *FLI1*-KO or *FLI1*-KO + *IDO1*-OE MC38 cells were inoculated into C57BL/6 mice, and tumors were dissected at day 21. The proportions of $CD4^+$ T cells among $CD45^+$ T cells **(J)**, and $CD25^+$ $FoxP3^+$ cells among $CD4^+$ T cells **(K)** isolated from the indicated tumors were determined by flow cytometry. **(L and M)** WT or *FLI1*-KO MC38 cells were inoculated into C57BL/6 mice. The depleting antibodies against $CD8^+$ and $CD4^+$ T cells were administered intraperitoneally every 3 days. The tumor growth rate **(L)** and endpoint tumor weight **(M)** are reported. Data (n = 5) shown are mean ± SD. Statistical significance was determined using one-way ANOVA **(J and K),** two-way ANOVA with Tukey multiple comparisons test **(L)** and two-tailed unpaired t test **(M)**. Source data are provided as a Source Data file.

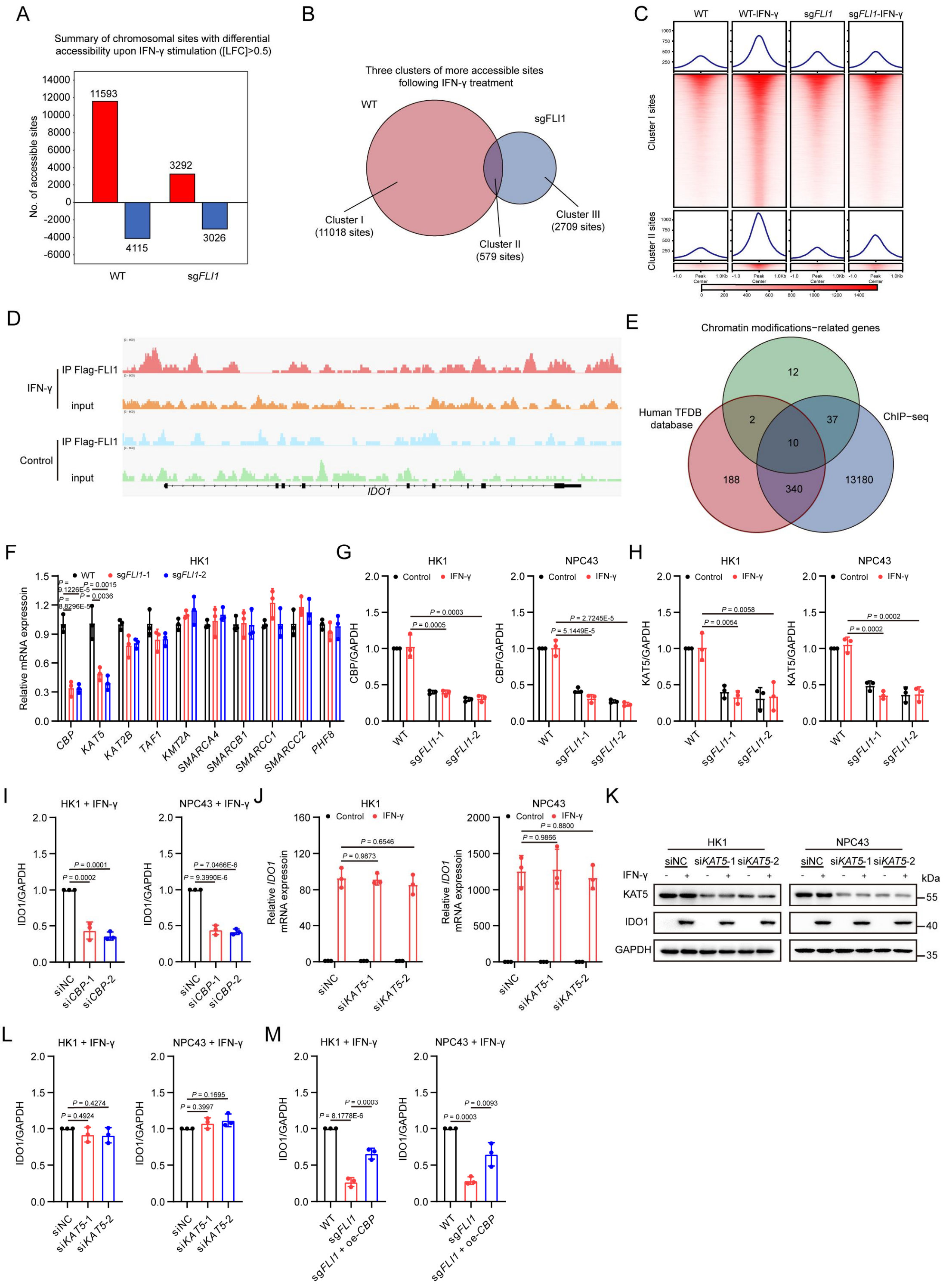

Supplementary Figure 3. (Extended data related to Figure 3)

(A and B) Genome-wide analysis **(A)** and Venn diagram **(B)** showed differentially accessible chromatin sites (|LFC| > 0.5) after IFN-γ stimulation in WT and *FLI1*-KO HK1 cells. **(C)** Chromatin accessibility heatmaps of WT and *FLI1*-KO HK1 cells. The heatmaps revealed the chromatin sites in cluster I and cluster II. Aggregated peak intensity within 1 kb center of chromatin regions with

differential accessibility is depicted. **(D)** Representation of Flag-FLI1 ChIP-seq and input profiles at the IDO1 gene locus in HK1 cells overexpressing Flag-FLI1, treated with or without IFN-γ. **(E)** Venn diagram displayed the overlap between the human TFDB database, chromatin modifications-related genes and Chip-seq data. **(F)** The mRNA levels of the indicated genes in *FLI1*-KO and WT HK1 cells were measured. **(G-H)** Effect of *FLI1* KO on CBP **(G)** and KAT5 **(H)** protein expression in HK1 and NPC43 cells, with or without IFN-γ. **(I)** Effect of *CBP* knockdown on IDO1 protein expression in HK1 and NPC43 cells upon IFN-γ stimulation. **(J-L)** IDO1 mRNA **(J)** and protein **(K and L)** levels in *KAT5*-knockdown and control HK1 and NPC43 cells upon IFN-γ stimulation were detected. **(M)** Effect of *CBP* OE on IDO1 protein expression in *FLI1*-KO HK1 and NPC43 cells, following IFN-γ treatment for 24 hours. The results are representative of three independent experiments **(F-M)**. The data are presented as the mean ± SD. Statistical analysis was performed by one-way ANOVA with Tukey multiple comparisons test **(F-J, L and M)**. Source data are provided as a Source Data file.

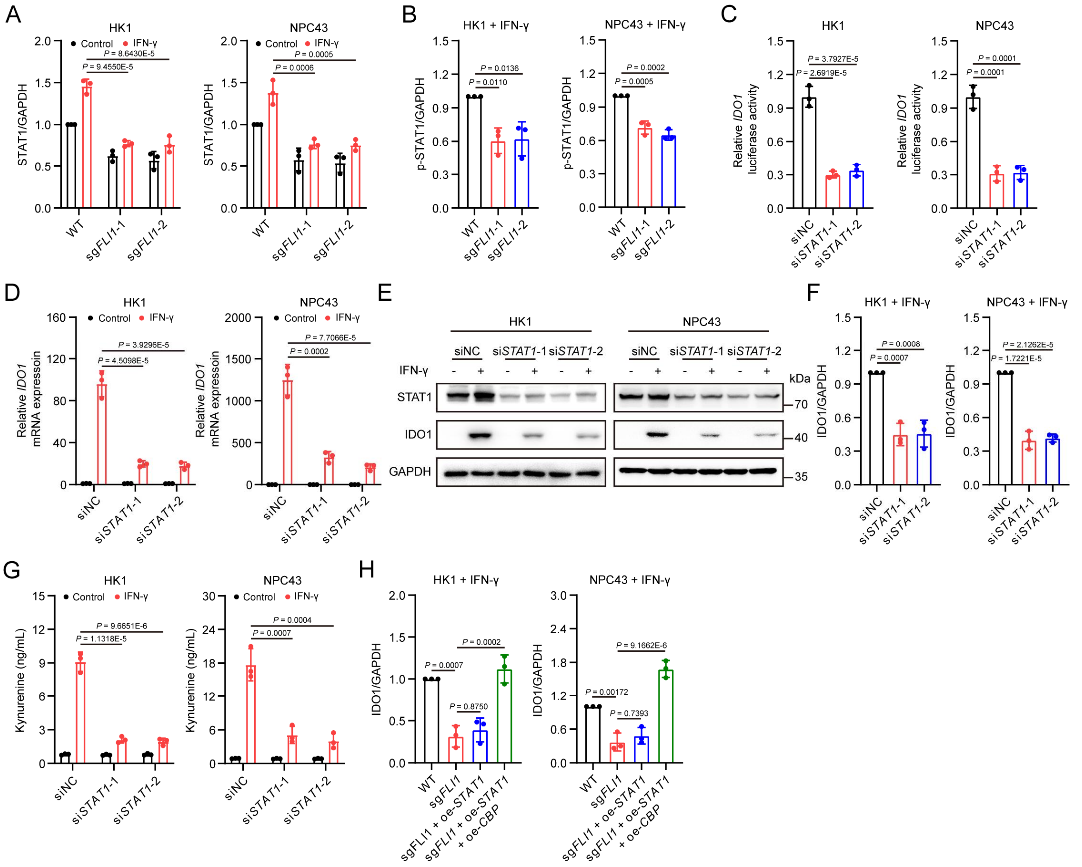

Supplementary Figure 4. (Extended data related to Figure 4)

(A) Effect of *FLI1* KO on STAT1 protein expression in HK1 and NPC43 cells, with or without IFN-γ. **(B)** Effect of *FLI1* KO on p-STAT1 protein expression in HK1 and NPC43 cells upon IFN-γ stimulation. **(C)** *STAT1*-knockdown and control HK1 and NPC43 cells were transfected with a *IDO1* promoter-luciferase reporter PGL4 plasmid for 24 h. Cells were then treated with IFN-γ for an additional 24h, followed by analysis of luciferase activity. **(D-F)** IDO1 mRNA **(D)** and protein **(E and F)** levels in *STAT1*-knockdown and control HK1 and NPC43 cells upon IFN-γ stimulation were detected. **(G)** Kyn production in the culture supernatants of the indicated NPC cells was determined. **(H)** Effect of *STAT1* OE with or without *CBP* OE on IDO1 protein expression in *FLI1*-KO HK1 and NPC43 cells upon IFN-γ stimulation. The results are representative of three independent experiments **(A-H)**. The data are presented as the mean ± SD. Statistical analysis was performed by one-way ANOVA with Tukey multiple comparisons test **(A-D and F-H)**. Source data are provided as a Source Data file.

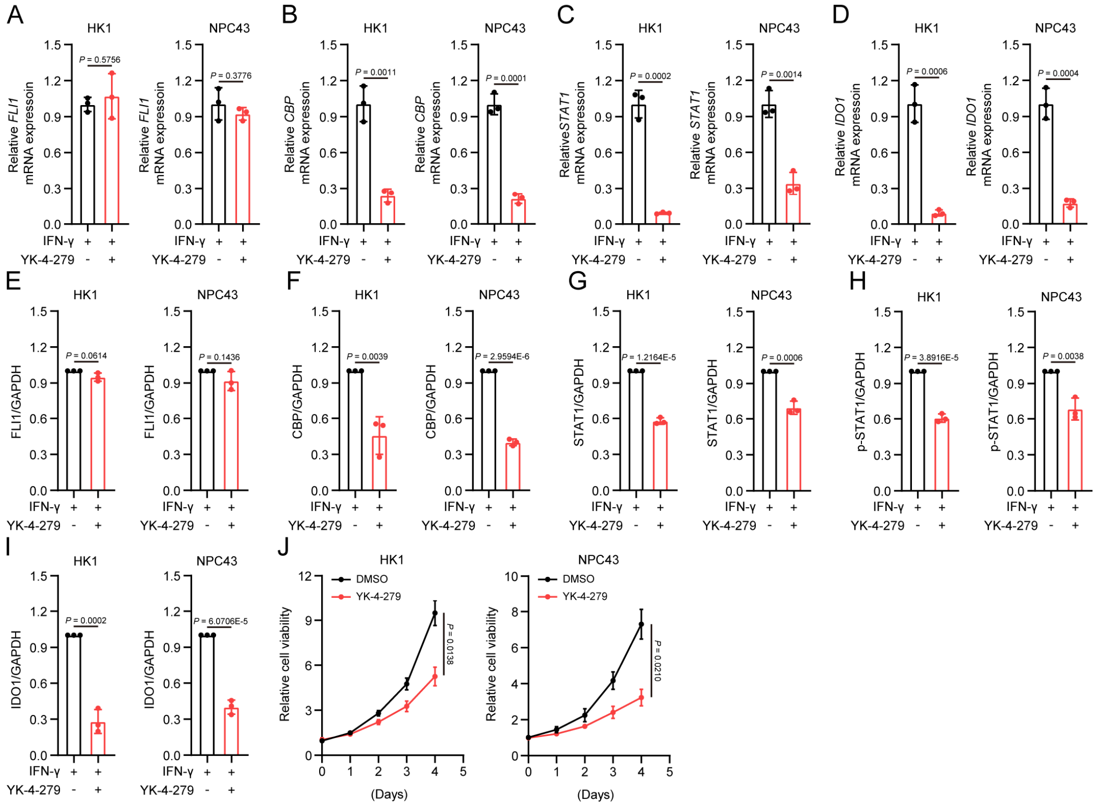

Supplementary Figure 5. (Extended data related to Figure 5)

(A-D) HK1 and NPC43 cells were pretreated with either DMSO or YK-4-279 for 24 hours, then IFN-γ was added for another 24 hours. The mRNA levels of *FLI1* **(A)**, *CBP* **(B)**, *STAT1* **(C)** and *IDO1* **(D)** in HK1 and NPC43 cells were determined. **(E-I)** Effect of YK-4-279 treatment on FLI1 **(E)**, CBP **(F)**, STAT1 **(G)**, p-STAT1 **(H)** and IDO1 **(I)** protein levels in HK1 and NPC43 cells upon IFN-γ stimulation. **(J)** The proliferation of HK1 and NPC43 cells treated with either DMSO or YK-4-279 was analyzed using the CCK-8 assays. The data are presented as the mean ± SD of three independent experiments **(A-J)**. Statistical analysis was performed by two-tailed unpaired *t* test **(A-I)** and two-way ANOVA **(J)**. Source data are provided as a Source Data file.

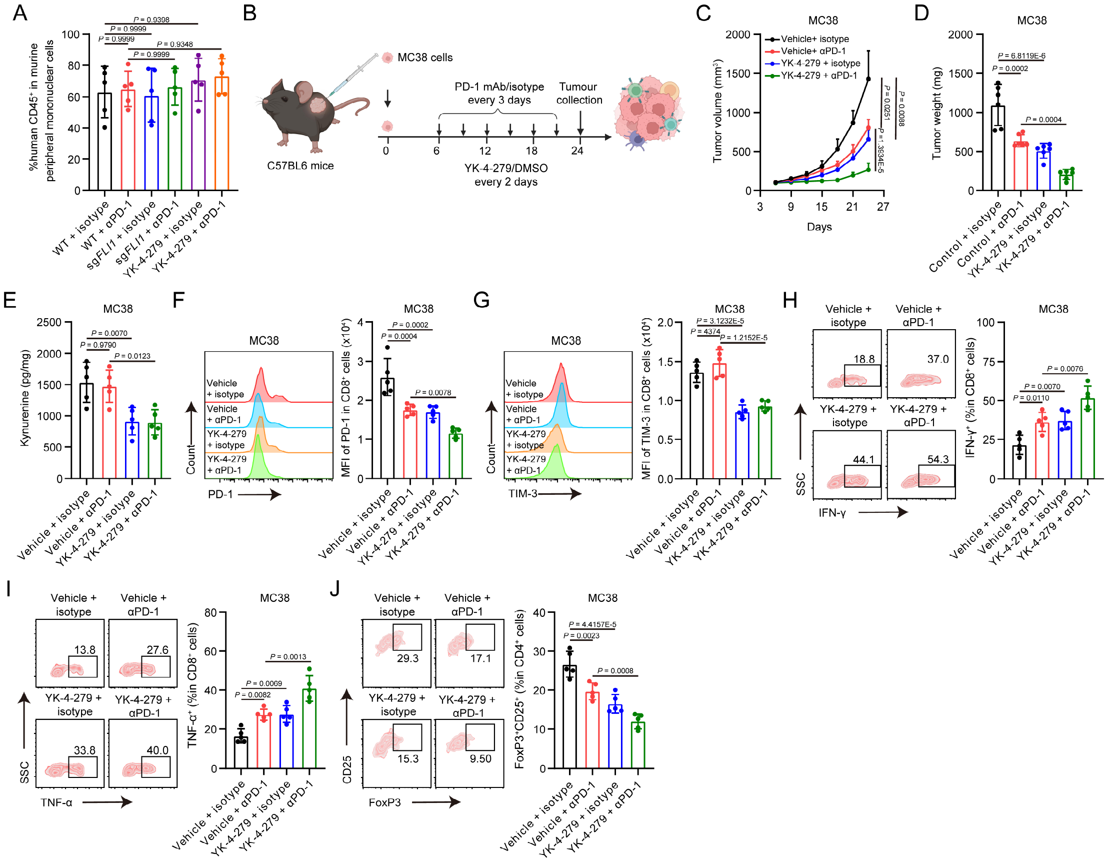

Supplementary Figure 6. (Extended data related to Figure 6)

(A) Percentage of human CD45$^+$ cells among peripheral mononuclear cells in humanized NSG mice ($n = 5$ in each group). **(B)** Schematic of tumor inoculation and treatment in mice. **(C and D)** Analysis of the tumor growth rate **(C)** and the endpoint tumor weight **(D)** ($n = 6$ in each group). **(E)** The level of Kyn in tumors was assessed by HPLC-MS ($n = 5$ in each group). **(F-I)** The expression of PD-1 **(F)** and TIM-3 **(G)** on CD8$^+$ T cells, and the expression of IFN-γ **(H)** and TNF-α **(I)** in CD8$^+$ T cells isolated from the indicated tumors were measured by flow cytometry ($n = 5$ in each group). **(J)** The proportion of CD25$^+$ FoxP3$^+$ cells among CD4$^+$ T cells isolated from the indicated tumors were determined by flow cytometry ($n = 5$ in each group). The data are presented as the mean ± SD **(A and C-J)**. Statistical analysis was performed by two-way ANOVA **(C)** and one-way ANOVA with Tukey multiple comparisons test **(A and D-J)**. Source data are provided as a Source Data file.

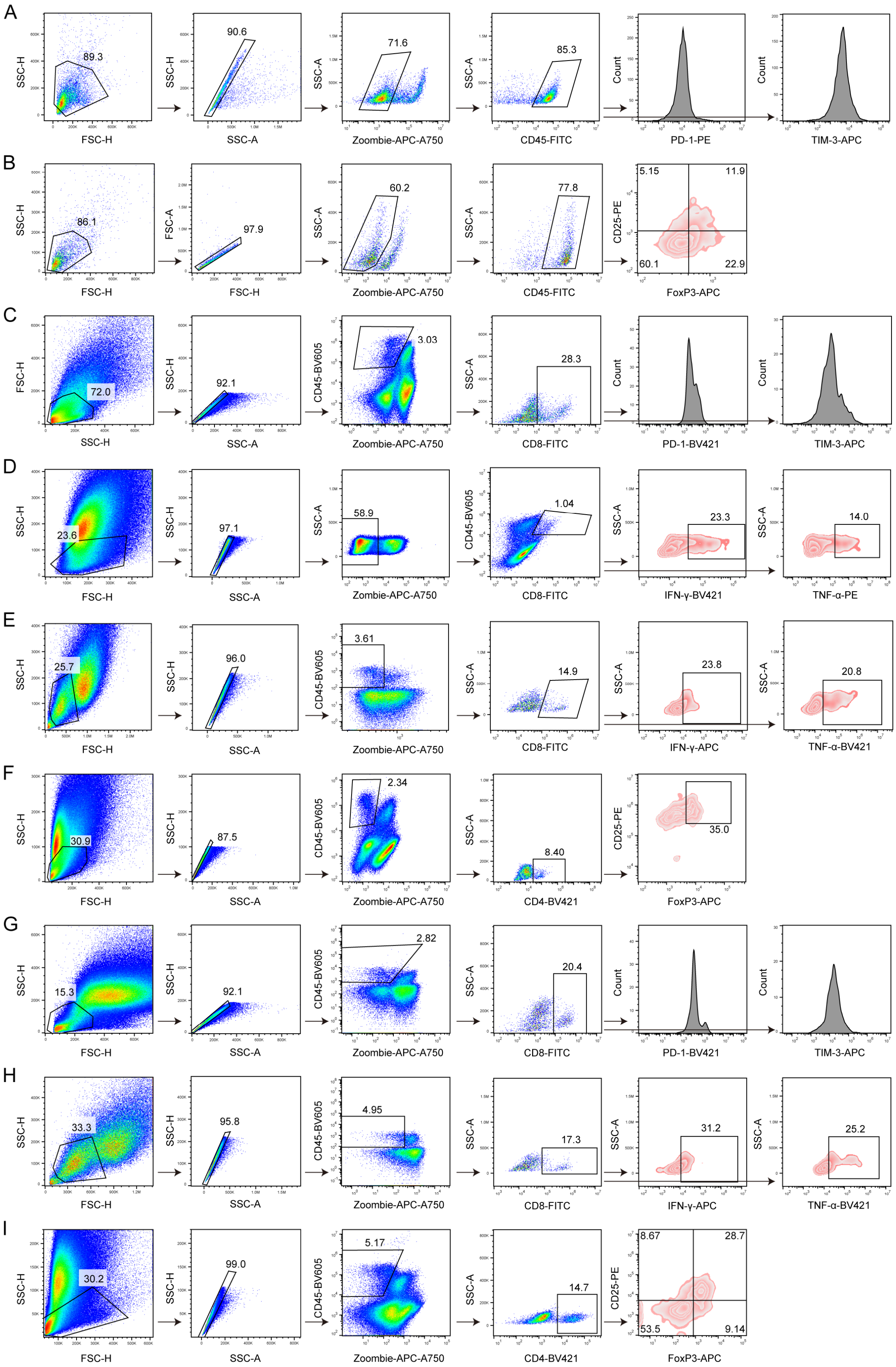

Supplementary Figure 7. (Gating strategy for flow cytometric analysis)

(A) Gating strategy for the flow cytometric analysis in Figures 1B, 1C, 2G, 2H, 5F and 5G.

(B) Gating strategy for the flow cytometric analysis in Figure S2I and 5J.

(C) Gating strategy for the flow cytometric analysis in Figures 1J, 1K, 2O, 2P, S6F and S6G.

(D) Gating strategy for the flow cytometric analysis in Figures 1L, 1M, S6H and S6I.

(E) Gating strategy for the flow cytometric analysis in Figures 2Q and 2R.

(F) Gating strategy for the flow cytometric analysis in Figure S2K and S6J.

(G) Gating strategy for the flow cytometric analysis in Figures 6F and 6G.

(H) Gating strategy for the flow cytometric analysis in Figures 6H and 6I.

(I) Gating strategy for the flow cytometric analysis in Figure 6J.

Supplementary Table 1. Correlations between FLI1-IDO1 expression level and clinical features in patients with nasopharyngeal carcinoma. The chi-square (χ^2) test was used to compare clinical characteristics.

Characteristic	Low expression group n=48 (100%)	High expression group n=25 (100%)	*P* value
Age			
≤47	27 (56.3)	16 (64.0)	0.523
>47	21 (43.8)	9 (36.0)	
Gender			
Male	33 (68.8)	17 (68.0)	0.948
Female	15 (31.3)	8 (32.0)	
TNM stage			
I-II	29 (60.4)	12 (48.0)	0.310
III-IV	19 (39.6)	13 (52.0)	
WHO type			0.250
IIa	7 (14.6)	1 (4.0)	
IIb	41 (85.4)	24 (96.0)	

Supplementary Table 2. List of Oligonucleotides used in this study.

**Gene**	**Sense**	**Antisense**
**RT-qPCR primers**		
FLI1	ACGGAAGTGCTGTTGTCACACC	CAAGCTCCTCTTCTGACTGAGTC
GAPDH	GTCTCCTCTGACTTCAACAGCG	ACCACCCTGTTGCTGTAGCCAA
IDO1	GCCTGATCTCATAGAGTCTGGC	TGCATCCCAGAACTAGACGTGC
CBP	AGTAACGGCACAGCCTCTCAGT	CCTGTCGATACAGTGCTTCTAGG
STAT1	ATGGCAGTCTGGCGGCTGAATT	CCAAACCAGGCTGGCACAATTG
IRF1	GAGGAGGTGAAAGACCAGAGCA	TAGCATCTCGGCTGGACTTCGA
SPI1	GACACGGATCTATACCAACGCC	CCGTGAAGTTGTTCTCGGCGAA
IKZF1	GCTGCCACAACTACTTGGAAAGC	AGTCTGTCCAGCACGAGAGATC
CYP1A1	GATTGAGCACTGTCAGGAGAAGC	ATGAGGCTCCAGGAGATAGCAG
CYP1B1	GCCACTATCACTGACATCTTCGG	CACGACCTGATCCAATTCTGCC
KAT5	GGAACTCACCACATTGCCTGTC	CTCATTGCCTGGAGGATGTCGT
KAT2B	GCACCATCTCAACGAAGACTGC	GTGTGGTTTCGTACCGAGGTAG
TAF1	GGCTAAAGCTCTGCGCTGACTT	AGCACTGCTCTGGTGACACCAT
KMT2A	GTGCTTTGTGGTCAGCGGAAGT	TGTGAGACAGCAACCCACGGTG
SMARCA4	CAAAGACAAGCACATCCTCGCC	GCCACATAGTGCGTGTTGAGCA
SMARCB1	GGCATCAGAAGACCTACGCCTT	CTCCATCTCAGCGTCTGTCAGA
SMARCC1	GAGAATGGACTGAACAGGAGACC	GGGTCCTCAATGGGAAGTCTCA
SMARCC2	CACTCGTGAGTGGACAGAACAG	GCAAGATGCACTCGTCCTGTGT
PHF8	GGACACATACAGTCATCAGGCAC	GGCTCTCATTTCCATCAAGGTCC
IDO1 promoter	agtgagacaccttgcctg	ccacttttcatagggaagaagtgg
CBP promoter	aggagctgggaacacgca	tttccagagcctgaacggg
STAT1 promoter	tgcaaacacagcacgtcct	CTCTGCGCAGGAAAGCGAA

**siRNA targeting sequence**		
siCBP-1	GCAAACAUCAGUGGGAAUUTT	AAUUCCCACUGAUGUUUGCTT
siCBP-2	CCAUUUCUCCUUCCCGAAUTT	AUUCGGGAAGGAGAAAUGGTT
siKAT5-1	GCCUCAAGCCGUGGUACUUTT	AAGUACCACGGCUUGAGGCTT
siKAT5-2	CCGUAGUCUCAAGUGUCUUTT	AAGACACUUGAGACUACGGTT
siSTAT1-1	CUCAGUGGUACGAACUUCATT	UGAAGUUCGUACCACUGAGTT
siSTAT1-2	GCUGGAUGAUCAAUAUAGUTT	ACUAUAUUGAUCAUCCAGCTT
**sgRNA targeting sequence**		
sgFLI1-1 (human)	CCCGTAGTCAGGACTCCCCG	CGGGGAGTCCTGACTACGGG
sgFLI1-2 (human)	TGATTGATCCACTCCTGCTG	CAGCAGGAGTGGATCAATCA
sgFLI1 (mouse)	AGTGAGAGTCAATGTCAAGC	GCTTGACATTGACTCTCACT

Supplementary Table 3. List of antibodies used in this study.

Antibodies used for western blot (WB), chromatin immunoprecipitation (ChIP) and immunohistochemistry (IHC)

Primary antibodies	Supplier	Catalogue No.	Application	Host species	Species activity	Dilution
anti-GAPDH	Proteintech	60004-1-Ig	WB	Mouse	Hu, Mo	1:5000
anti-FLI1	Abcam	ab133485	WB, IHC	Rabbit	Hu, Mo	1:2000 for WB, 1:200 for IHC
anti-IDO1	Cell Signaling Technology	86630S	WB, IHC	Rabbit	Hu	1:2000 for WB, 1:200 for IHC
anti-CBP	Abcam	ab253202	WB	Rabbit	Hu, Mo	1:2000
anti-STAT1	Cell Signaling Technology	14994S	WB	Rabbit	Hu, Mo	1:2000
anti-Phospho-Stat1 (Tyr701)	Cell Signaling Technology	9167S	WB	Rabbit	Hu, Mo	1:2000
anti-KAT5	Proteintech	10827-1-AP	WB	Rabbit	Hu, Mo	1:2000
anti-tagFLAG	Proteintech	20543-1-AP	ChIP	Rabbit	Hu, Mo	5ug per test
anti-H3K9Ac	Active Motif	61251	ChIP	Mouse	Hu, Mo	5ug per test
anti-H3K14Ac	Active Motif	39697	ChIP	Rabbit	Hu	5ug per test
anti-H3K18Ac	Active Motif	39755	ChIP	Rabbit	Hu	5ug per test
anti-H3K27Ac	Active Motif	39133	ChIP	Rabbit	Hu	5ug per test
anti-cleaved Caspase-3	Cell Signaling Technology	9664S	IHC	Rabbit	Hu, Mo	1:200
anti-Ki-67	Cell Signaling Technology	28074-1-AP	IHC	Rabbit	Hu, Mo	1:200

Antibodies used for flow cytometric (FC) analysis

Primary antibodies	Supplier	Catalogue No.	Application	Host species	Species activity	clone	Dilution
Brilliant Violet 605™ anti-mouse CD45	Biolegend	103139	FC	Rabbit	Mo	30-F11	1:100
FITC anti-mouse CD8a	Biolegend	100706	FC	Rabbit	Mo	53-6.7	1:100

Brilliant Violet 421™ anti-mouse PD-1	Biolegend	135217	FC	Rabbit	Mo	29F.1A12	1:100
APC anti-mouse TIM-3	Biolegend	119706	FC	Rabbit	Mo	RMT3-23	1:100
Brilliant Violet 421™ anti-mouse IFN-γ	Biolegend	505829	FC	Rabbit	Mo	XMG1.2	1:100
PE anti-mouse TNF-α	Biolegend	506306	FC	Rabbit	Mo	MP6-XT22	1:100
Brilliant Violet 421™ anti-mouse CD4	Biolegend	100437	FC	Rabbit	Mo	GK1.5	1:100
PE anti-mouse CD25	Biolegend	102008	FC	Rabbit	Mo	PC61	1:100
Alexa Fluor® 647 anti-mouse/rat/human FOXP3	Biolegend	320014	FC	Mouse	Hu, Mo, Rat	150D	1:100
FITC anti-human CD45	Biolegend	304006	FC	Mouse	Hu	HI30	1:100
PE anti-human PD-1	Biolegend	329906	FC	Mouse	Hu	EH12.2H7	1:100
APC anti-human TIM-3	Biolegend	345012	FC	Mouse	Hu	F38-2E2	1:100
PE anti-human CD25	Biolegend	302606	FC	Mouse	Hu	BC96	1:100
Brilliant Violet 605™ anti-human CD45	Biolegend	304042	FC	Mouse	Hu	HI30	1:100
FITC anti-human CD8a	Biolegend	300906	FC	Mouse	Hu	HIT8a	1:100
Brilliant Violet 421™ anti-human PD-1	Biolegend	329920	FC	Mouse	Hu	EH12.2H7	1:100
APC anti-human IFN-γ	Biolegend	502512	FC	Mouse	Hu	4S.B3	1:100
Brilliant Violet 421™ anti-human TNF-α	Biolegend	502932	FC	Mouse	Hu	MAb11	1:100
Brilliant Violet 421™ anti-human CD4	Biolegend	300532	FC	Mouse	Hu	RPA-T4	1:100
PE anti-human CD25	Biolegend	302606	FC	Mouse	Mo	BC96	1:100
APC anti-mouse IFN-γ	Biolegend	505810	FC	Rabbit	Mo	XMG1.2	1:100
Brilliant Violet 421™ anti-mouse TNF-α	Biolegend	506328	FC	Rabbit	Mo	MP6-XT22	1:100
