# Supplementary material for: FLI1 promotes IFN-γ-induced kynurenine production to impair anti-tumor immunity
Source: Nat Commun. 2024 May 30;15:4590. doi: 10.1038/s41467-024-48397-9 (PMC11139667; doi:10.1038/s41467-024-48397-9)
Supplement: Supplementary file 3 — Reporting summary [file 41467_2024_48397_MOESM3_ESM.pdf]

nature portfolio

Corresponding author(s): Wuguo Deng

Last updated by author(s): Mar 26, 2024

Reporting Summary

Nature Portfolio wishes to improve the reproducibility of the work that we publish. This form provides structure for consistency and transparency in reporting. For further information on Nature Portfolio policies, see our Editorial Policies and the Editorial Policy Checklist.

Statistics

For all statistical analyses, confirm that the following items are present in the figure legend, table legend, main text, or Methods section.

n/a	Confirmed	
☐	☒	The exact sample size (*n*) for each experimental group/condition, given as a discrete number and unit of measurement
☐	☒	A statement on whether measurements were taken from distinct samples or whether the same sample was measured repeatedly
☐	☒	The statistical test(s) used AND whether they are one- or two-sided<br>*Only common tests should be described solely by name; describe more complex techniques in the Methods section.*
☒	☐	A description of all covariates tested
☐	☒	A description of any assumptions or corrections, such as tests of normality and adjustment for multiple comparisons
☐	☒	A full description of the statistical parameters including central tendency (e.g. means) or other basic estimates (e.g. regression coefficient) AND variation (e.g. standard deviation) or associated estimates of uncertainty (e.g. confidence intervals)
☐	☒	For null hypothesis testing, the test statistic (e.g. *F*, *t*, *r*) with confidence intervals, effect sizes, degrees of freedom and *P* value noted<br>*Give P values as exact values whenever suitable.*
☒	☐	For Bayesian analysis, information on the choice of priors and Markov chain Monte Carlo settings
☒	☐	For hierarchical and complex designs, identification of the appropriate level for tests and full reporting of outcomes
☐	☒	Estimates of effect sizes (e.g. Cohen's *d*, Pearson's *r*), indicating how they were calculated

Our web collection on statistics for biologists contains articles on many of the points above.

Software and code

Policy information about availability of computer code

Data collection	FASC: cytoFLEX LX; Real-time PCR: Bio-Rad CFX96; LC/MS: Q-Exactive mass spectrometer.
Data analysis	Image Lab version 4.1 (Bio-Rad) was used to acquire Immunoblots and protein Coomassie staining gels. GraphPad Prism version 9 and spss27 were used for data analysis; FACS data were analyzed with FlowJo version 10; Pathways gene enrichment were analyzed by using GSEA v4.3.2; Immunohistochemical Scoring were performed using HALO version 3.2.1851 (Indica Labs); Raw LC/MS data was analyzed with MZmine 2.5.3; ssGSEA analysis was performed with R program version 4.0.3 (R Foundation for Statistical Computing); JASPAR software was used to predict the FLI1-binding motif.

For manuscripts utilizing custom algorithms or software that are central to the research but not yet described in published literature, software must be made available to editors and reviewers. We strongly encourage code deposition in a community repository (e.g. GitHub). See the Nature Portfolio guidelines for submitting code & software for further information.

Data

Policy information about availability of data

All manuscripts must include a data availability statement. This statement should provide the following information, where applicable:

- Accession codes, unique identifiers, or web links for publicly available datasets
- A description of any restrictions on data availability
- For clinical datasets or third party data, please ensure that the statement adheres to our policy

The raw sequence data of RNA-seq, ChIP-seq and ATAC-seq data generated in this study have been deposited in the GEO database under accession code GSE247898 (https://www.ncbi.nlm.nih.gov/geo/query/acc.cgi?acc=GSE247898). TCGA datasets for COAD, READ, LUSC and SKCM, OV, STAD and BLCA were all obtained from UCSC Xena [https://xenabrowser.net/datapages/]. The NPC sequencing data used in this study are available in the GEO database under accession code GSE102349 (https://www.ncbi.nlm.nih.gov/geo/query/acc.cgi?acc=GSE102349). The core transcription factor gene set was obtained from the JASPAR database (https://jaspar.elixir.no/). The remaining data generated in this study are provided in the Supplementary Information/Source Data file. Source data are provided with this paper.

Research involving human participants, their data, or biological material

Policy information about studies with human participants or human data. See also policy information about sex, gender (identity/presentation), and sexual orientation and race, ethnicity and racism.

Reporting on sex and gender	The gender information of the patients with nasopharyngeal carcinoma were retrospectively collected through the medical record.
Reporting on race, ethnicity, or other socially relevant groupings	The relevent information of patients with nasopharyngeal carcinoma were retrospectively collected through the medical record.
Population characteristics	The patients' clinical characteristics are listed in Supplementary Table 1.
Recruitment	We collected 10 NPC samples for mRNA expression analysis from the Sun Yat-sen University Cancer Center (Guangzhou, China),and collected 110 paraffin-embedded NPC samples from Shanghai Outdo Biotech company (Shanghai, China) over the time period from 2010 to 2011 for survival analysis.
Ethics oversight	The Institutional Ethical Review Boards of Sun Yat-sen University Cancer Center approved this study (B2023-232-01), in which anonymized data were analysed, and waived the requirement for informed consent.<br>Informed consent was obtained from all patients and approved by the research medical ethics committee of Shanghai Outdo Biotech company (Approval Number: SHYJS-CP-1704009).

Note that full information on the approval of the study protocol must also be provided in the manuscript.

Field-specific reporting

Please select the one below that is the best fit for your research. If you are not sure, read the appropriate sections before making your selection.

☒ Life sciences ☐ Behavioural & social sciences ☐ Ecological, evolutionary & environmental sciences

For a reference copy of the document with all sections, see nature.com/documents/nr-reporting-summary-flat.pdf

Life sciences study design

All studies must disclose on these points even when the disclosure is negative.

Sample size	No statistical method was used to predetermine sample size. Sample size was chosen based on previous experience and standards in the field (KLi, N., Quan, A., Li, D. et al. Nat Commun 14, 1986 (2023); or LWang, Y., Zhou, SK., Wang, Y. et al. Nat Commun 14, 1993 (2023).). For example, all of the experiments were repeated at least 3 times, and the sample sizes for in vitro (n >= 3) and in vivo (n >= 5) .
Data exclusions	No data were excluded from the analyses.
Replication	As reported in the figure legends, experiments were performed at least three times with similar results, the findings were reliably reproduced.
Randomization	The samples used in this study were randomly allocated into control or experimental groups.
Blinding	The investigators were not blinded to sample allocation during experiment and outcome assessment, because results used were obtained using objective quantitative methods.

Reporting for specific materials, systems and methods

We require information from authors about some types of materials, experimental systems and methods used in many studies. Here, indicate whether each material, system or method listed is relevant to your study. If you are not sure if a list item applies to your research, read the appropriate section before selecting a response.

Materials & experimental systems

n/a	Involved in the study
☐	☒ Antibodies
☐	☒ Eukaryotic cell lines
☒	☐ Palaeontology and archaeology
☐	☒ Animals and other organisms
☒	☐ Clinical data
☒	☐ Dual use research of concern
☒	☐ Plants

Methods

n/a	Involved in the study
☐	☒ ChIP-seq
☐	☒ Flow cytometry
☒	☐ MRI-based neuroimaging

Antibodies

Antibodies used

Antibodies used for western blot (WB), chromatin immunoprecipitation (ChIP) and immunohistochemistry (IHC)
anti-GAPDH, Proteintech, Cat# 60004-1-Ig, WB, 1:5000
anti-FLI1, Abcam, Cat# ab133485, WB, IHC, 1:2000 for WB, 1:200 for IHC
anti-IDO1, Cell Signaling Technology, Cat# 86630S, WB, IHC, 1:2000 for WB, 1:200 for IHC
anti-CBP, Abcam, Cat# ab253202, WB, 1:2000
anti-STAT1, Cell Signaling Technology, Cat# 14994S, WB, 1:2000
anti-Phospho-Stat1 (Tyr701), Cell Signaling Technology, Cat# 9167S, WB, 1:2000
anti-KAT5, Proteintech, Cat# 10827-1-AP, WB, 1:2000
anti-tagFLAG, Proteintech, Cat# 20543-1-AP, ChIP, 5ug per test
anti-H3K9Ac, Active Motif, Cat# 61251, ChIP, 5ug per test
anti-H3K14Ac, Active Motif, Cat# 39697, ChIP, 5ug per test
anti-H3K18Ac, Active Motif ,Cat# 39755, ChIP, 5ug per test
anti-H3K27Ac, Active Motif, Cat# 39133, ChIP, 5ug per test
anti-cleaved Caspase-3, Cell Signaling Technology, Cat# 9664S, IHC, 1:200
anti-Ki-67 Cell Signaling Technology Cat# 28074-1-AP, IHC, 1:200

Antibodies used for flow cytometric (FC) analysis (dilution 1:100 for all antibodies)
Brilliant Violet 605TM anti-mouse CD45, Biolegend, Cat# 103139, Clone 30-F11
FITC anti-mouse CD8a, Biolegend, Cat# 100706, Clone 53-6.7
Brilliant Violet 421™ anti-mouse PD-1, Biolegend, Cat# 135217, Clone 29F.1A12
APC anti-mouse TIM-3, Biolegend, Cat# 119706, Clone RMT3-23
Brilliant Violet 421™ anti-mouse IFN-γ, Biolegend, Cat# 505829, Clone XMG1.2
PE anti-mouse TNF-α Biolegend, Cat# 506306, Clone MP6-XT22
Brilliant Violet 421™ anti-mouse CD4, Biolegend, Cat# 100437, Clone GK1.5
PE anti-mouse CD25, Biolegend, Cat# 102008, Clone PC61
Alexa Fluor® 647 anti-mouse/rat/human FOXP3, Biolegend, Cat# 320014, Clone 150D
FITC anti-human CD45, Biolegend, Cat# 304006, Clone HI30
PE anti-human PD-1, Biolegend, Cat# 329906, Clone EH12.2H7
APC anti-human TIM-3, Biolegend, Cat# 345012, Clone F38-2E2
PE anti-human CD25, Biolegend, Cat# 302606, Clone BC96
Brilliant Violet 605TM anti-human CD45, Biolegend, Cat# 304042, Clone HI30
FITC anti-human CD8a, Biolegend, Cat# 300906, Clone HIT8a
Brilliant Violet 421™ anti-human PD-1, Biolegend, Cat# 329920, Clone EH12.2H7
APC anti-human IFN-γ, Biolegend, Cat# 502512, Clone 4S.B3
Brilliant Violet 421™ anti-human TNF-α, Biolegend, Cat# 502932, Clone MAb11
Brilliant Violet 421™ anti-human CD4, Biolegend, Cat# 300532, Clone RPA-T4
PE anti-human CD25, Biolegend, Cat# 302606, Clone BC96
APC anti-mouse IFN-γ, Biolegend, Cat# 505810, Clone XMG1.2
Brilliant Violet 421™ anti-mouse TNF-α, Biolegend, Cat# 506328, Clone MP6-XT22

Validation

anti-GAPDH, Proteintech, Cat# 60004-1-Ig. Supplier validation: "Species reactivity, Reacts with: Human, Mouse, Rat, Yeast, Plant, Zebrafish, Pig, applications suitable for: FC, IF, IP, WB, ELISA".
anti-FLI1, Abcam, Cat# ab133485. Supplier validation: "Species reactivity, Reacts with: Human, Mouse, applications suitable for: IHC-P, WB".
anti-IDO1, Cell Signaling Technology, Cat# 86630S. Supplier validation: "Species reactivity, Reacts with: Human, applications suitable for: IHC, WB, IP, IF, F".
anti-CBP, Abcam, Cat# ab253202. Supplier validation: "Species reactivity, Reacts with: Mouse, Rat, Human, applications suitable for: IHC-P, WB, ICC/IF, ChIP, Flow Cyt (Intra), ChIC/CUT&RUN-seq, IP".
anti-STAT1, Cell Signaling Technology, Cat# 14994S. Supplier validation: "Species reactivity, Reacts with: Mouse, Rat, Human,

applications suitable for: IHC, WB, IF, F, ChIP, IP".
anti-Phospho-Stat1 (Tyr701), Cell Signaling Technology, Cat# 9167S. Supplier validation: "Species reactivity, Reacts with: Mouse, Human, applications suitable for: IHC, WB, IF, F, ChIP, CUT&RUN-seq, IP".
anti-KAT5, Proteintech, Cat# 10827-1-AP. Supplier validation: "Species reactivity, Reacts with: Human, Mouse, Rat, applications suitable for: IHC, WB, ELISA".
anti-tagFLAG, Proteintech, Cat# 20543-1-AP. Supplier validation: "Species reactivity, Reacts with: Human, Mouse, Rat, Chicken, Yeast, Monkey, Pig, Duck, applications suitable for: ChIP, CoIP, FC, IF, IHC, IP, RIP, WB".
anti-H3K9Ac, Active Motif, Cat# 61251. Supplier validation: "Species reactivity, Reacts with: Human, Mouse, applications suitable for: ChIP, ICC/IF, IHC, WB".
anti-H3K14Ac, Active Motif, Cat# 39697. Supplier validation: "Species reactivity, Reacts with: Human, applications suitable for: ChIP, WB".
anti-H3K18Ac, Active Motif ,Cat# 39755. Supplier validation: "Species reactivity, Reacts with: Human, applications suitable for: ChIP, ICC/IF, DB, WB".
anti-H3K27Ac, Active Motif, Cat# 39133. Supplier validation: "Species reactivity, Reacts with: Budding Yeast, Human, applications suitable for: ChIP, ICC/IF, WB, CUT&Tag".
anti-cleaved Caspase-3, Cell Signaling Technology, Cat# 9664S. Supplier validation: "Species reactivity, Reacts with: Human, Rabbit, Mouse, Monkey, applications suitable for: WB, IP, IHC, IF, F".
anti-Ki-67 Cell Signaling Technology Cat# 28074-1-AP. Supplier validation: "Species reactivity, Reacts with: Human, Rat, Mouse, applications suitable for: FC, IF, IHC, ELISA".
Brilliant Violet 605TM anti-mouse CD45, Biolegend, Cat# 103139. Supplier validation: "Verified Reactivity Mouse, Application FC - Quality tested".
FITC anti-mouse CD8a, Biolegend, Cat# 100706. Supplier validation: "Verified Reactivity Mouse, Application FC - Quality tested".
Brilliant Violet 421™ anti-mouse PD-1, Biolegend, Cat# 135217. Supplier validation: "Verified Reactivity Mouse, Application FC - Quality tested".
APC anti-mouse TIM-3, Biolegend, Cat# 119706. Supplier validation: "Verified Reactivity Mouse, Application FC - Quality tested".
Brilliant Violet 421™ anti-mouse IFN-γ, Biolegend, Cat# 505829. Supplier validation: "Verified Reactivity Mouse, Application ICFC - Quality tested - Quality tested".
PE anti-mouse TNF-α Biolegend, Cat# 506306. Supplier validation: "Verified Reactivity Mouse, Application ICFC- Quality tested".
Brilliant Violet 421™ anti-mouse CD4, Biolegend, Cat# 100437. Supplier validation: "Verified Reactivity Mouse, Application FC - Quality tested, ICC - Verified".
PE anti-mouse CD25, Biolegend, Cat# 102008. Supplier validation: "Verified Reactivity Mouse, Application FC - Quality tested".
Alexa Fluor® 647 anti-mouse/rat/human FOXP3, Biolegend, Cat# 320014. Supplier validation: "Verified Reactivity Human, Mouse, Rat, Application ICFC - Quality tested".
FITC anti-human CD45, Biolegend, Cat# 304006. Supplier validation: "Verified Reactivity Human, Application FC - Quality tested".
PE anti-human PD-1, Biolegend, Cat# 329906. Supplier validation: "Verified Reactivity Human, Application FC - Quality tested".
APC anti-human TIM-3, Biolegend, Cat# 345012. Supplier validation: "Verified Reactivity Human, Application FC - Quality tested".
PE anti-human CD25, Biolegend, Cat# 302606. Supplier validation: "Verified Reactivity Human, Application FC - Quality tested".
Brilliant Violet 605TM anti-human CD45, Biolegend, Cat# 304042. Supplier validation: "Verified Reactivity Human, Application FC - Quality tested".
FITC anti-human CD8a, Biolegend, Cat# 300906. Supplier validation: "Verified Reactivity Human, Application FC - Quality tested".
Brilliant Violet 421™ anti-human PD-1, Biolegend, Cat# 329920. Supplier validation: "Verified Reactivity Human, Application FC - Quality tested".
APC anti-human IFN-γ, Biolegend, Cat# 502512. Supplier validation: "Verified Reactivity Human, Application ICFC - Quality tested".
Brilliant Violet 421™ anti-human TNF-α, Biolegend, Cat# 502932. Supplier validation: "Verified Reactivity Human, Application ICFC - Quality tested".
Brilliant Violet 421™ anti-human CD4, Biolegend, Cat# 300532. Supplier validation: "Verified Reactivity Human, Application ICFC - Quality tested, ICC - Verified".
PE anti-human CD25, Biolegend, Cat# 302606. Supplier validation: "Verified Reactivity Human, Application FC - Quality tested".
APC anti-mouse IFN-γ, Biolegend, Cat# 505810. Supplier validation: "Verified Reactivity Mouse, Application ICFC - Quality tested".
Brilliant Violet 421™ anti-mouse TNF-α, Biolegend, Cat# 506328. Supplier validation: "Verified Reactivity Mouse, Application ICFC - Quality tested".

Eukaryotic cell lines

Policy information about cell lines and Sex and Gender in Research

Cell line source(s)

The human nasopharyngeal epithelial cell line NP69 and NPC cell lines HK1, C666-1 were provided by Professor Musheng Zeng at Sun Yat-sen University Cancer Center (Guangzhou). The human NPC cell lines NPC43, NPC38 and NPC53 were purchased from the Nasopharyngeal Carcinoma (NPC) Tissue Bank. HEK293T and MC38 cells were purchased from the American Type Culture Collection (ATCC).

Authentication

Cell lines were authenticated by short tandem repeat (STR) fingerprinting.

Mycoplasma contamination

All cell lines were tested negative for mycoplasma contamination.

Commonly misidentified lines
(See ICLAC register)

None.

Animals and other research organisms

Policy information about studies involving animals; ARRIVE guidelines recommended for reporting animal research, and Sex and Gender in Research

Laboratory animals

6-week-old female C57BL/6 mice were procured from the Guangdong Medical Laboratory Animal Center (Foshan, China). A 6-week-

Laboratory animals	old female, SPF humanized NSG mouse model (Shanghai Model Organisms) was created through the administration of 5 x 106 human peripheral blood mononuclear cells (PBMCs) via tail vein injection. Mice were housed in temperature-controlled pathogen-free conditions (around 20?, 40% humidity) under a 12-h light/dark cycle.
Wild animals	The study did not involve wild animals.
Reporting on sex	All animals used in this study were female, and no sex-based analysis was performed.
Field-collected samples	The study did not involve samples collected from the field.
Ethics oversight	Animal experiments in this study were approved by the Experimental Animal Ethics Committee, Sun Yat-sen University Cancer Center (L102042023050F).

Note that full information on the approval of the study protocol must also be provided in the manuscript.

Plants

Seed stocks	*Report on the source of all seed stocks or other plant material used. If applicable, state the seed stock centre and catalogue number. If plant specimens were collected from the field, describe the collection location, date and sampling procedures.*
Novel plant genotypes	*Describe the methods by which all novel plant genotypes were produced. This includes those generated by transgenic approaches, gene editing, chemical/radiation-based mutagenesis and hybridization. For transgenic lines, describe the transformation method, the number of independent lines analyzed and the generation upon which experiments were performed. For gene-edited lines, describe the editor used, the endogenous sequence targeted for editing, the targeting guide RNA sequence (if applicable) and how the editor was applied.*
Authentication	*Describe any authentication procedures for each seed stock used or novel genotype generated. Describe any experiments used to assess the effect of a mutation and, where applicable, how potential secondary effects (e.g. second site T-DNA insertions, mosiacism, off-target gene editing) were examined.*

ChIP-seq

Data deposition

☒ Confirm that both raw and final processed data have been deposited in a public database such as GEO.

☒ Confirm that you have deposited or provided access to graph files (e.g. BED files) for the called peaks.

Data access links<br>*May remain private before publication.*	The ChIP-seq data reported in this paper have been deposited in the Gene Expression Omnibus (GEO) database (https://www.ncbi.nlm.nih.gov/geo/query/acc.cgi?acc=GSE247898).
Files in database submission	Raw sequencing files: Sample_R22016288-22C0028-22C0028_R1.fastq.gz, Sample_R22016288-22C0028-22C0028_R2.fastq.gz, Sample_R22016280-22C0029P-22C0029P_R1.fastq.gz, Sample_R22016280-22C0029P-22C0029P_R2.fastq.gz, Sample_R22016283-22C0028P-22C0028P_R1.fastq.gz, Sample_R22016283-22C0028P-22C0028P_R2.fastq.gz, Sample_R22016276-22C0029-22C0029_R1.fastq.gz and Sample_R22016276-22C0029-22C0029_R2.fastq.gz
Genome browser session<br>(e.g. UCSC)	Not available.

Methodology

Replicates	No technical replicates.
Sequencing depth	All ChIP-seq datasets were sequenced with 2*150bp paired end, each experimental total reads 9G.
Antibodies	Flag Antibody (Proteintech, China)
Peak calling parameters	We performed peak calling by using MACS.
Data quality	Data quality for ChIP-seq was assessed with Qiagen QC metrics.
Software	Bowtie2 and MACS.

Flow Cytometry

Plots

Confirm that:

☒ The axis labels state the marker and fluorochrome used (e.g. CD4-FITC).

☒ The axis scales are clearly visible. Include numbers along axes only for bottom left plot of group (a 'group' is an analysis of identical markers).

☒ All plots are contour plots with outliers or pseudocolor plots.

☒ A numerical value for number of cells or percentage (with statistics) is provided.

Methodology

Sample preparation	The sample preparation was described in the methods section.
Instrument	All data were obtained with a CYTOFLEX flow cytometer (Beckman Coulter).
Software	The results were analysed using Flow Jo software 10.
Cell population abundance	Minimum of 3,000 cells were counted for each analysis.
Gating strategy	The gating strategy were provided in the Supplementary Figure 7.

☒ Tick this box to confirm that a figure exemplifying the gating strategy is provided in the Supplementary Information.
